# Screening of Trauma Patients in Need of Knee Radiography by Triage Nurses and Using Ottawa Knee Rule; a Letter to Editor 

**Published:** 2019-10-06

**Authors:** Samad Shams Vahdati, Alireza Ala, Zhila Samani, Sasan Ghazanfar Ahari, Mohammad Mirza-Aghazadeh-Attari

**Affiliations:** 1 Emergency Medicine Research Team, Tabriz University of Medical Sciences, Tabriz, Iran.; 2 Emergency Medicine Department, Tabriz University of Medical Sciences, Tabriz, Iran.; 3 Student Research committee, Tabriz University of Medical Sciences, Tabriz, Iran.; 4 Aging Research Institute, Tabriz University of Medical Sciences, Tabriz, Iran.; 5 Medical Radiation Sciences Research Group, Department of Radiology, Imam Reza Hospital, Tabriz University of Medical Sciences, Tabriz, Iran.

**Keywords:** Multiple Trauma, decision support techniques, emergency nursing, triage, diagnostic imaging

**Dear Editor**

In some clinical guidelines, emergency triage nurses request radiographies according to the clinical decision-making priorities. While some studies have suggested that nurses are not as accurate as doctors in assessing major complications of trauma to the knee, it is thought that they are as capable in detecting minor traumas and inconsequential episodes (1). Many studies have shown that the cost of treatment and the waiting period for patients in the emergency department decrease without missing fractures when traumas are initially assessed by a certified nurse. Routinely, a clinical criteria termed Ottawa Knee Rule is utilized to determine if a patient is in need of a knee radiography or not (2, 3). According to this guideline, if a patient with suspected knee trauma meets any of the following criteria, he or she should undergo imaging modalities: Age above 55 years, tenderness at head of fibula, isolated tenderness of patella, inability to flex the knee more than 90°, and inability to bear weight (4). 

**Table 1:** Frequency of different radiographic findings

**Radiographic finding **	**Number (%) **
Femoral shaft fracture	52 (23.6)
Fibula fracture	44 (20.0)
Tibia fracture	24 (10.9)
Tibia & fibula fracture	12 (5.5)
Pelvic fracture	12 (5.5)
Intertrochanteric fracture	4 (1.8)
Femoral fracture with pelvic fracture	4 (1.8)
Femoral fracture with tibia fracture	4 (1.8)
Patella fracture	4 (1.8)
Fracture of the leg with humerus fracture	4 (1.8)
No fracture	56 (25.5)

**Table 2:** Comparison of Ottawa knee rule and triage levels by emergency nurses and physicians regarding the screening of patients in need of knee radiography

**Variables**	**Physicians**	**Nurses**	**P**
**Ottawa knee variables**			
Age ≥55	68 (30.9)	68 (30.9)	NA
Isolated patellar tenderness	104 (47.3)	104 (47.3)	NA
Tenderness at the fibular head	76 (41.8)	94 (42.8)	0.048
Unable to flex knee to 90°	160 (72.7)	152 (69.1)	0.231
Unable to bear weight	208 (94.5)	216 (98.2)	0.036
**Triage level**			
Level one	8 (3.6)	8 (3.6)	0.632
Level two	164 (74.6)	172 (78.2)	
Level three	48 (21.8)	40(18.2)	
**Screening characteristics**			
True positive	164	164	NA
True negative	12	5	
False positive	44	51	
False negative	0	0	
Sensitivity	100 (97.1–00.0)	100 (97.1–100)	NA
Specificty	21.4 ( 12.0 – 34.7)	8.9 (3.3 – 20.3)	0.030
PPV	78.8 (72.5– 84.0)	76.2 (69.9–81.6)	0.643
NPV	100 (69.9–100)	100 (46.3–100)	NA
PLR	3.7 (2.7–4.8)	3.2 (2.5–4.1)	0.043
NLR	0.0	0.0	NA
Total accuracy	80.0 (74.1 – 58.1)	77.5 (71.5–82.8)	0.640

Data are presented as frequency (%). NA: not applicable. PPV: positive predictive value; NPV: negative predictive value; PLR: positive likelihood ratio; NLR: negative likelihood ratio.

Investigating the ability of emergency nursing staff in triage of patients in need of knee radiography, the authors of this article selected 238 trauma patients who were admitted to a tertiary referral trauma center from March 2018 to October 2018, using a random number generator. 

Triage nurses evaluated the patients using Ottawa knee rule and recorded their triage level. Then, all selected cases were assessed by an emergency physician and the level of triage regarding knee trauma was recorded, again. Finally, the patient's knee radiographs were taken, and the findings of nurses and physicians were compared. 

A five-hour course was conducted to train the theory and practice of Ottawa knee rule to triage nurses. They were provided with a pocket flowchart that helped them be alert during triage. The knee radiographs were obtained by a single machine and interpreted by Radiology residents (years 2-4). Patients with decreased level of consciousness (Glasgow coma scale below 13) or multiple trauma, < 8 years old, with unstable vital signs, and not willing to participate in the study, were excluded. The Data were analyzed using SPSS software version 15.00.

Finally, 18 patients were excluded due to lack of consent for taking part in the study or being discharged against medical advice, and 220 patients with the mean age of 43.94 ± 20.44 (8 – 95) years were triaged (74.5 % male). The most common trauma mechanism was pedestrian accident with 21.8%, followed by motorcycle accident 18.2%. Table 1 depicts the results of radiographies obtained from the patients. 

The results of triage levels by emergency physicians and triage nurses are presented in table 2. The records of the two groups were significantly different regarding tenderness at the fibular head and inability to bear weight (p <0.05). 

It should be noted that, despite the 100% sensitivity of the rule in identifying the patients in need of knee radiography (both by physicians and emergency nurses), sensitivity of the test was very low (21.4% by physicians and 8.9% by emergency nurses). This means that a considerable numbers of cases (20% to 23% of cases) underwent diagnostic imaging and limb radiation without indication. 

In conclusion, it seems that further training is needed before use of Ottawa knee rule by emergency triage nurses in routine triage of trauma patients. 
